# Citrulline protects mice from experimental cerebral malaria by ameliorating hypoargininemia, urea cycle changes and vascular leak

**DOI:** 10.1371/journal.pone.0213428

**Published:** 2019-03-08

**Authors:** Irene Gramaglia, Joyce Velez, Yu-Sun Chang, Wilson Caparros-Wanderley, Valery Combes, Georges Grau, Monique F. Stins, Henri C. van der Heyde

**Affiliations:** 1 La Jolla Infectious Disease Institute, San Diego, CA, United States of America; 2 Chang-Gung University, Taipei, Taiwan; 3 WCW Biostatistical Consulting, Aylesbury, United Kingdom; 4 School of Life Sciences, University of Technology Sydney, Sydney, Australia; 5 Vascular Immunology Unit, University of Sydney, Sydney, Australia; 6 Johns Hopkins Bloomberg School of Public Health, Baltimore, MD, United States of America; Université Pierre et Marie Curie, FRANCE

## Abstract

Clinical and model studies indicate that low nitric oxide (NO) bioavailability due in part to profound hypoargininemia contributes to cerebral malaria (CM) pathogenesis. Protection against CM pathogenesis may be achieved by altering the diet before infection with *Plasmodium falciparum* infection (nutraceutical) or by administering adjunctive therapy that decreases CM mortality (adjunctive therapy). This hypothesis was tested by administering citrulline or arginine in experimental CM (eCM). We report that citrulline injected as prophylaxis immediately post infection (PI) protected virtually all mice by ameliorating (i) hypoargininemia, (ii) urea cycle impairment, and (iii) disruption of blood brain barrier. Citrulline prophylaxis inhibited plasma arginase activity. Parasitemia was similar in citrulline- and vehicle control-groups, indicating that protection from pathogenesis was not due to decreased parasitemia. Both citrulline and arginine administered from day 1 PI in the drinking water significantly protected mice from eCM. These observations collectively indicate that increasing dietary citrulline or arginine decreases eCM mortality. Citrulline injected ip on day 4 PI with quinine-injected ip on day 6 PI partially protected mice from eCM; citrulline plus scavenging of superoxide with pegylated superoxide dismutase and pegylated catalase protected all recipients from eCM. These findings indicate that ameliorating hypoargininemia with citrulline plus superoxide scavenging decreases eCM mortality.

## Introduction

A hallmark of blood-stage *Plasmodium falciparum* (Pf) infection in humans is the development of profound hypoargininemia leading to impaired nitric oxide (NO) bioavailability as measured by reactive hyperemia [1–4]. Both hypoargininemia and impaired NO bioavailability are higher in severe malaria (SM) patients than uncomplicated malaria (UM), suggesting hypoargininemia contributes to impaired NO bioavailability that, in turn, contributes to malarial pathogenesis [2]. Indeed, infusions of arginine into UM and moderately ill Pf-infected patients are well tolerated and restored the reactive hyperemia responses in these patients [2, 3, 5]. Other factors including free hemoglobin in plasma and low tetrahydrobiopterin may exacerbate low NO bioavailability in Pf-infected patients because free hemoglobin is a potent scavenger of NO, and tetrahydrobiopterin is a cofactor for nitric oxide synthase (NOS) enzyme that converts arginine to citrulline and NO[6–9].

Experimental cerebral malaria (eCM) studies support the conclusion that low NO bioavailability contributes to malarial pathogenesis [10–12]. Low NO bioavailability in eCM is caused by hypoargininemia, free hemoglobin, and low tetrahydrobiopterin levels [10, 13–15]. NO donor-injection ip and inhaled NO from day 1 PI onwards protect against eCM mortality, and disruption of the blood brain barrier (BBB) during eCM [10, 15]. NO donor injection also provides partial protection from eCM mortality when injected late in the infection as adjunctive therapy together with anti-parasite chemotherapy [15].

Unfortunately, the above findings have not translated into an effective adjunctive therapy for CM [16, 17]. Infusion of low dose arginine into SM patients does not restore NO bioavailability and inhaled NO therapy does not affect CM mortality but inhaled NO decreases neurocognitive impairment [16, 17]. High levels of free arginase in SM patients may convert infused arginine to ornithine preventing its use as a substrate for endothelial NOS (eNOS) [2]. Infused citrulline offers advantages over arginine in that (i) infused arginine activates arginase enzyme, and (ii) citrulline inhibits arginase, resulting in improved maintenance of blood arginine levels by citrulline infusion [18, 19]. A second factor potentially preventing the restoration of NO bioavailability after arginine infusion is the production of NO-scavenging superoxide (SO) during malaria due to uncoupled eNOS [11, 14]. We therefore tested whether (i) arginine/citrulline administered as a nutraceutical protects against eCM, (ii) citrulline is more effective than arginine at ameliorating eCM as an adjunctive therapy, and (iii) scavenging SO in conjunction with amelioration of hypoargininemia provides improved protection over amelioration of hypoargininemia alone.

## Methods

### Ethics statement

The Institutional Animal Care and Use Committee of La Jolla Infectious Disease Institute approved all protocols and procedures.

### Mouse studies

There are differences between humans and mice and between *Plasmodium* species infecting humans and those infecting mice, indicating caution is needed in extrapolating results to humans [20]. Nevertheless, the key CM hallmarks under investigation (hypoargininemia, superoxide [SO] production, and disruption of the BBB) appear to be conserved in human and eCM [21].

For eCM, we injected iv groups of C57BL/6 mice (6–10 weeks old; Jackson Laboratories) with 1×10^5^ RBCs infected with *Plasmodium berghei* ANKA (PbA-iRBCs; MR4) in 0.2ml of PBS. The inoculum was prepared from a source mouse as described [10, 22]. Between 200 and 1000 RBCs were counted in Giemsa-stained thin blood films from experimental animals to assess parasitemia. To assess the extent of eCM, *Pb*A-infected mice were given neurological tests daily from day 4PI onwards. These tests comprised the sum of the righting reflex and gripping reflex each on a scale of 1–5 with 5 exhibiting no impairment [10, 22]. Animals with a score of <4 were moribund and euthanized.

*Treatments*. Citrulline or arginine (Millipore-Sigma, St. Louis, MO) was injected i.p. in 0.8ml saline (430 mM/mouse) twice daily. For nutraceutical studies, the injection was initiated at day 1PI through day 12 PI or added to the drinking water on day 1PI. Arginine or citrulline were added to the drinking water at 731mM of amino acid. A mouse’s average daily consumption is 3mls of water per mouse per day. For the adjunctive therapy studies, citrulline was injected ip twice daily starting on day 4PI followed by administration of anti-parasite chemotherapy, quinine i.p (120mg/Kg; Millipore-Sigma, St. Louis, MO). Artemisinin rather than quinine is the standard of care for human malaria because artemisinin kills both ring and later stages whereas quinine is not effective against ring-stages [23]. The use of quinine likely does not affect our conclusions about whether citrulline or arginine function as adjunctive therapy because the mechanism(s) of protection are likely independent of the anti-parasitic agent.

*Analytical methods*. Arginase activity was measured by colorimetric assay kit (Millipore-Sigma, St. Louis, MO). The Evans Blue dye extrusion experiment was performed as described in [22, 24]. PET imaging performed by University of California San Diego Imaging Core facility was performed with normalization for decay FDG during the course of the experiment as described in [25]. Thrombocytopenia was measured by flow cytometry on 1μl of tail vein blood as described by us [26, 27]. Selected inflammatory and coagulation were measured by bead array with standards for each molecule by using Rules Based Medicine [10]. The GSSG:GSH ratio and D2-labeled citrulline and arginine levels were measured by mass spectrometry (Metabolon Inc,Morrisville, NC and Chang-Gung Mass Spectrometry Core Facility, respectively).

#### Statistical analysis

Analysis of variance with the Prism program (GraphPad) with Tukey’s post-hoc test was performed to statistically compare all measurements with a *P* value cut-off of 0.05. The mean and standard error of the mean of the results are reported in text and figures. Survival curves are compared with non-parametric Logrank test with a *P* value cut-off of 0.05 with the “neurological period” for the development of eCM occurring day 6 to 12 in C57BL/6 mice.

## Results

### Citrulline as a nutraceutical protects against eCM

#### P. berghei infection elicits hypoargininemia, hypocitrullinemia, and hyperornithinemia

The urea cycle mediates removal of toxic ammonia and recycles citrulline back into arginine. (S1A Fig). Arginine is converted into eCM-protective NO by nitric oxide synthase (NOS) but may also be converted into ornithine by arginase [10] (S1A Fig). ypoarginemia is reported during eCM^9^, which may be due to elevated activity of arginase or NOS. We therefore measured the levels of the selected amino acids of the urea cycle on day 0 (uninfected), 4 (patent) and 6 PI with *P*. *berghei*. Plasma arginine declined markedly (>10 fold) on day 4 PI and declined further by day 6 PI [10]. Plasma ornithine levels were increased only on day 4 PI and then decreased on day 6 PI compared with day 4 PI (S1B Fig). Plasma citrulline declined slightly by day 4 PI with a ~50% decrease on day 6 PI (S1B Fig). These results confirm early hypoargininemia and indicate late hypocitrullinemia with transient elevation of plasma ornithine that coincides with onset of hypoargininemia during eCM.

#### Citrulline restores plasma arginine levels after infusion on day 5PI of P. berghei infection for >2h and is converted to arginine with significantly lower levels of ornithine compared to arginine infusion

**Fig 1:**
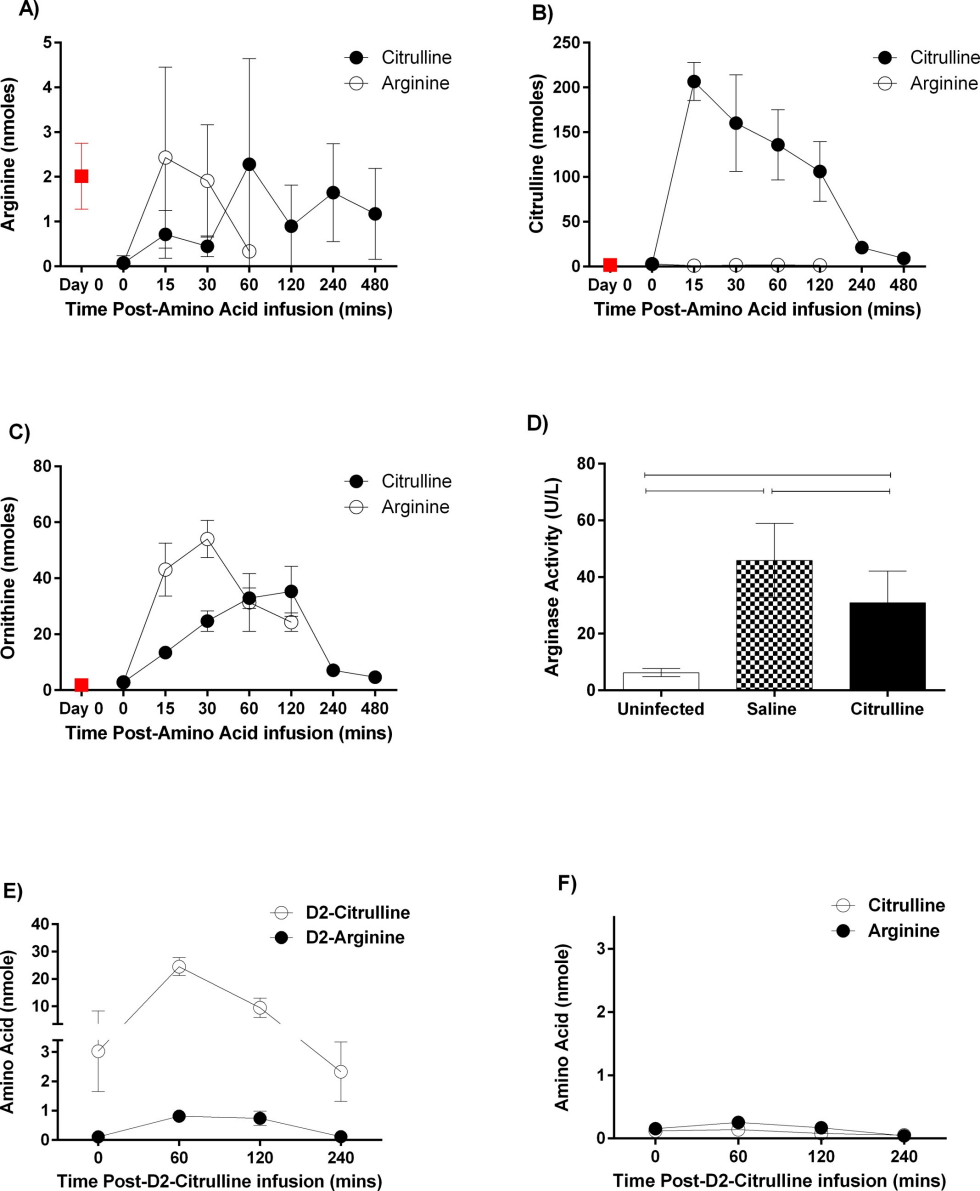
Citrulline is converted to arginine on day 5 of eCM restoring plasma arginine levels to uninfected levels. Mice were injected i.v. with PbA on day 0 PI. **A-C:** On day 5PI (just prior to eCM on day 6PI), mice were injected ip with equimolar citrulline or arginine (430 mM) at time 0 and then plasma from n = 3 mice was obtained at the selected timepoints. The plasma levels of (A) arginine, (B) citrulline, and (C) ornithine were measured by HPLC. Uninfected mice (n = 3) were control group. The experiment was repeated. Citrulline; Arginine. **D:** Mice were injected twice daily from day 1 PI with citrulline or saline (vehicle); uninfected, untreated mice were used as controls. On day 6PI, plasma was collected, and arginase activity was measure using the arginase activity assay (Millipore-Sigma, St-Louis,MO). p<0.05. Repeated i.p injections of arginine elicited pathology in the peritoneal cavity and were not performed again. This experiment was repeated for citrulline. **E, F**: Groups (n = 3) of mice were injected with D2-labeled citrulline and the levels of arginine and citrulline assessed by mass spectrometry at the selected timepoints. (E) D2-Citrulline; D2-Arginine; (F) Citrulline; Arginine.

To assess the time-course of plasma arginine, citrulline, and ornithine levels (major components of the urea cycle) following a single dose of arginine or citrulline, we injected ip equimolar doses of citrulline and arginine into groups (n = 3 total) of mice on day 5PI of *P*. *berghei* infection and assessed the plasma levels of amino acids by HPLC at 5 timepoints (Fig 1A–1C). Both citrulline and arginine injection rapidly (<15 min) restored plasma arginine levels (Fig 1A) but only citrulline injection raised plasma citrulline (Fig 1B). Arginine injection exhibited significantly increased levels of ornithine (p<0.05) compared with citrulline-injected group (Fig 1C), suggesting that infused arginine is converted by plasma arginase to ornithine at a lower rate, potentially allowing free arginine to be used NOS to produce eCM-protective NO.

To verify that injected citrulline is indeed converted into arginine, we injected D2-labeled citrulline into groups of mice on day 5 of eCM and measured the relative levels of labeled and unlabeled arginine and citrulline by mass spectrometry. The levels of both D2-arginine and D2-citrulline increased significantly (p<0.05) within 1h and then declined slowly over the course of 4hrs (Fig 1E); the levels unlabeled arginine and citrulline did not change markedly over the same time period (Fig 1F). These findings collectively indicate that infused citrulline is converted to arginine in animals just prior to eCM (day 6PI).

Citrulline has the additional benefit over arginine of inhibiting arginase [18], which may allow some plasma arginine to be converted to eCM-protective NO by NOS rather than to ornithine by arginase. To test whether this also occurs in eCM, we assessed the level of arginase activity in uninfected, citrulline and vehicle control treated mice on day 6 PI. Plasma from vehicle control mice exhibited significantly (p<0.05; ~3fold) higher arginase activity compared to uninfected mice; arginase activity was significantly (p<0.05; ~0.3fold) lower in citrulline nutraceutical group compared with vehicle controls (Fig 1D). Taken together, citrulline restores plasma arginine levels and inhibits plasma arginase, suggesting infused citrulline may be better than infused arginine for restoring plasma arginine for NOS.

#### Citrulline injection as a nutraceutical protects against eCM whereas equimolar arginine exhibits toxicity

**Fig 2:**
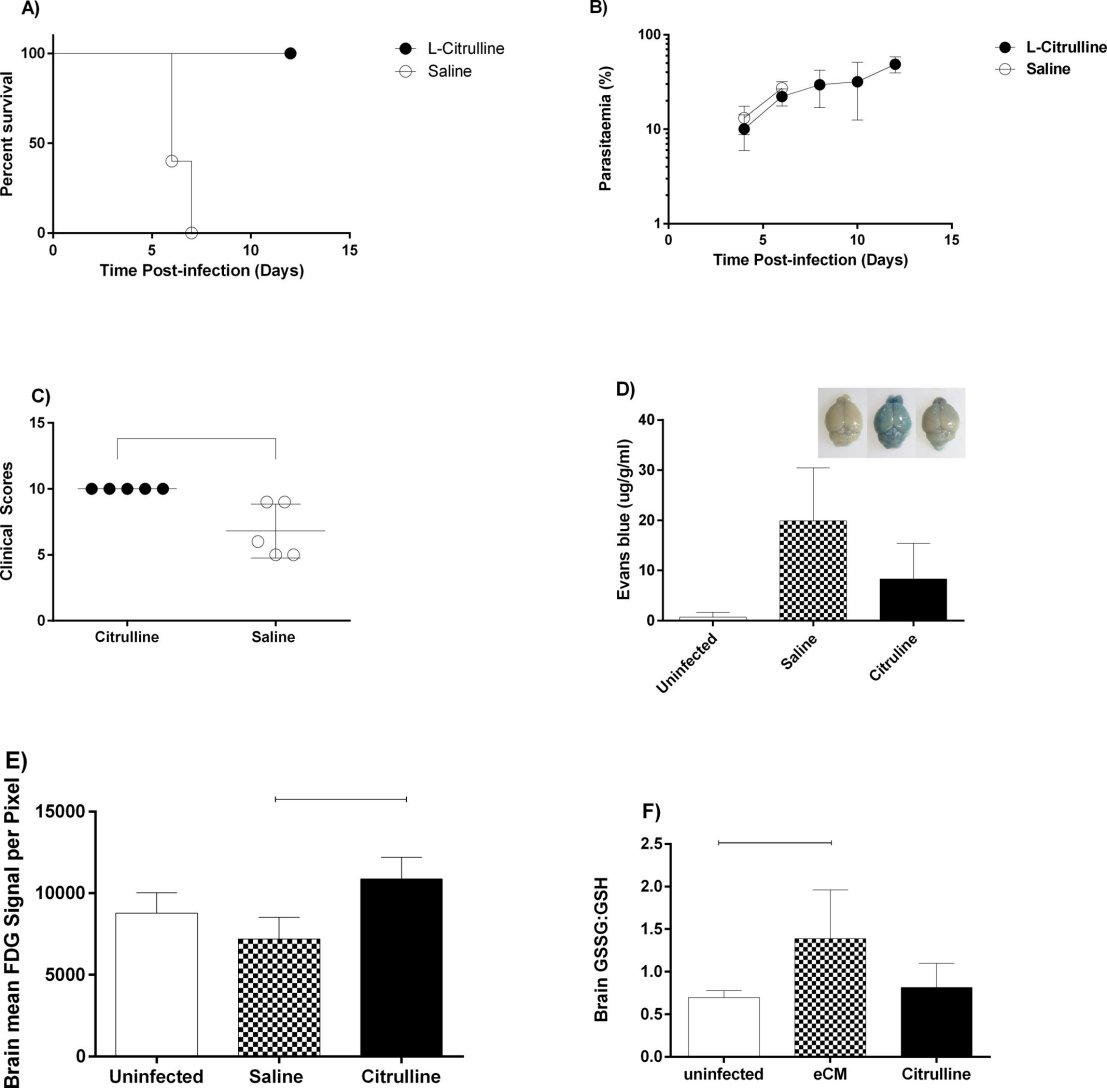
Citrulline as a nutraceutical protects against eCM. Groups (n = 5) of mice were injected i.v. with PbA and then injected twice daily ip with citrulline (nutraceutical) or saline. (**A):** survival, (**B):** parasitemia (mean % ± stderr) timecourse, and **(C)**: clinical scores of citrulline- and saline-injected mice infected with PbA. This experiment was repeated 4 times with similar results. Citrulline; Arginine **(D):** Vascular leak into brain measured by Evans Blue dye extrusion into brain parenchyma in groups (n = 5) of mice on day 6 PI (eCM) treated with citrulline as a nutraceutical or vehicle controls. Inset: representative images of brains after Evans Blue extrusion. This experiment was repeated with similar results. **(E)**: Average pixel intensity PET brain images in groups (n = 5) mice infected with PbA and treated with citrulline nutraceutical or saline and compared with uninfected controls. This experiment was performed once. : p<0.05. **(F)**: Brain GSSG:GSH ratios in groups (n = 5) of mice infected with PbA and treated with Citrulline or saline twice daily i.p and compared to uninfected, untreated controls. Analysis performed by Metabolon inc (Morrisville, NC) using mass spectrometry.

To determine whether arginine and citrulline as a nutraceutical protect against eCM, we injected ip groups of mice from day 1PI with equimolar arginine or citrulline. We discontinued arginine injections because of toxicity within the peritoneal cavity at the injection site. All citrulline-injected mice were significantly (p<0.05) protected from eCM mortality compared with vehicle controls, which all succumbed (Fig 2A); the differences in mortality were not attributable to changes in parasite replication because parasitemia was similar (p>0.05) in both groups (Fig 2B). The clinical scores were significantly (p<0.05) improved in citrulline-treated mice compared with saline-injected mice exhibiting eCM (Fig 2C). This result indicates that citrulline as prophylaxis is protective against eCM.

#### Citrulline as a nutraceutical during eCM inhibits arginase activity while maintaining (i) BBB integrity (vascular leak and hemorrhage), (ii) brain glucose consumption, (iii) urea cycle integrity, (iv) oxidative stress measured by GSSG:GSH ratio, (v) inflammatory molecules and (vi) thrombocytopenia

Hallmarks of eCM include the breakdown of the blood brain barrier (BBB), hypoargininemia (with disruption of urea cycle), inflammation, coagulopathy, and oxidative stress [12, 28, 29]. PET imaging measures glucose utilization and may identify brain cell impairment, which may occur in eCM brains. To determine whether citrulline nutraceutical protects against the above hallmarks of eCM, we performed a series of experiments in citrulline nutraceutical-treated animals and vehicle controls at eCM (day 6PI) compared with uninfected controls. Evans Blue dye extrusion through the vasculature into brain parenchyma is marked in eCM brains, indicating a marked breakdown of the BBB [22, 24]. The citrulline nutraceutical prophylaxed group of *P*. *berghei*-infected mice exhibited significantly (p<0.05; ~50%) decreased brain vascular leak compared with vehicle eCM controls (Fig 2D). The metabolic activity in brain was significantly (p<0.05) greater in citrulline nutraceutical prophylaxed group of *P*. *berghei*-infected mice compared with vehicle-injected eCM controls, which showed low metabolic activity (Fig 2E). In the liver, metabolic activity was higher in both nutraceutical and vehicle controls (S2E Fig), indicating increased metabolic activity in the liver that is not ameliorated by citrulline.

Arginine is a key component of the urea cycle and so hypoargininemia should affect this cycle (S1 Fig). Mice prophylaxed with citrulline nutraceutical the exhibited significant (p<0.05) hypercitrullinemia and hyperornithinemia on day 6 PI prior to citrulline injection, as well as markedly restored hypoargininemia, and lower glutamine levels (S2A–S2D Fig). The levels of some proinflammatory molecules measured by bead array (VCAM1, TNF, MIP3β, MMP9, lymphotactin, IL18, and MCSF1) were significantly (p<0.05) decreased in citrulline nutraceutical group on day 6 PI compared with saline injected eCM controls (S1 Table). Thrombocytopenia measured by flow cytometry [30] and selected coagulation molecules measured by the bead array[10] were similar (p>0.05) in citrulline nutraceutical group on day 6PI and saline controls with eCM (S3 Fig and S2 Table).

The GSSG:GSH ratio, measured by mass spectrometry, was significantly (p<0.05; ~1fold) elevated in saline injected eCM controls compared with uninfected controls. Citrulline nutraceutical prophylaxed mice exhibited significantly (p<0.05;~1fold) decreased GSSG:GSH ratio on day 6 PI compared with vehicle-injected eCM controls (Fig 2F), suggesting that citrulline may reduce oxidant-stress during eCM. In summary, citrulline nutraceutical prophylaxis ameliorated BBB breakdown, restored hypoargininemia, decreased oxidant stress, reduced proinflammatory cytokine levels and improved the brain metabolic activity compared with vehicle-injected eCM controls. This citrulline prophylaxis did not improve thrombocytopenia or levels of coagulation-molecules.

#### Oral citrulline and arginine in drinking water protect against eCM

**Fig 3:**
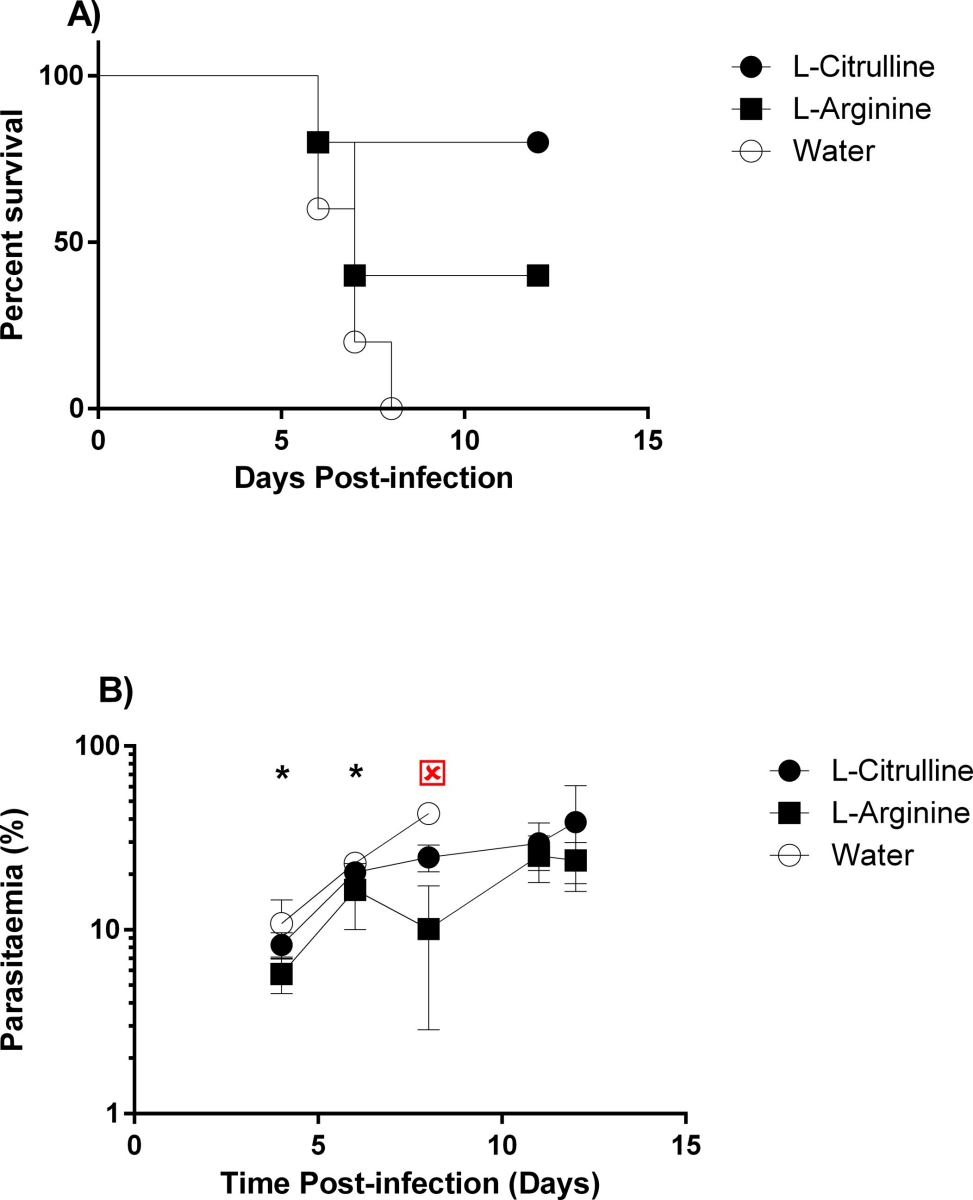
Both oral arginine and citrulline protect against eCM. Groups (n = 5) of mice were injected i.v. with PbA on day 0 PI and allowed to drink equimolar (731mM) arginine or citrulline ad libitum (~3ml/mouse). (**A):** survival, (**B):** parasitemia (mean % ± stderr) timecourse of arginine-, citrulline-, and water-treated mice infected with PbA. Citrulline; Arginine; Water. This experiment was repeated twice with similar results. In one replicate experiment, mice on arginine in drinking water exhibited significantly (p<0.05) lower parasitemia than water controls, but this effect was not consistently observed. : p<0.05 Arginine vs Water control;: p<0.05 Arginine vs Citrulline.

Injection ip permits precise control over dosing, but oral consumption is optimal for a nutraceutical. We infected the groups of mice with PbA and then added either citrulline or arginine to the drinking water daily from day 1PI. The citrulline and to a lesser extent arginine-prophylaxed mice were significantly (p<0.05) protected from eCM mortality compared with vehicle control (Fig 3A). However, citrulline ingestion did not affect parasite replication because the parasitemia was similar to the saline group, whereas arginine ingestion significantly decreased parasitemia (Fig 3B). This result indicates that citrulline and arginine consumed as a nutraceutical may protect against eCM. Additional studies are needed to determine whether this high dose can be achievable and effective in humans and whether lower doses function in eCM.

### Citrulline plus SO scavenging protects against eCM as adjunctive therapy

SO is produced during malaria and scavenges NO to produce peroxynitrite. The contribution of SO to eCM and malaria is questioned because pHox-deficient mice are not protected from eCM and exhibit similar parasitemia as wild-type controls [31–33]. To determine whether SO functions in eCM pathogenesis, we injected ip groups of mice daily from day 1PI with SO scavenger PEG-SOD+PEG-CAT or saline as a control. Mice injected with SO scavenger as prophylaxis were significantly (p<0.05) protected from eCM whereas all saline controls succumbed (S3A Fig). The parasitemia was similar in both groups (S3B Fig).

**Fig 4:**
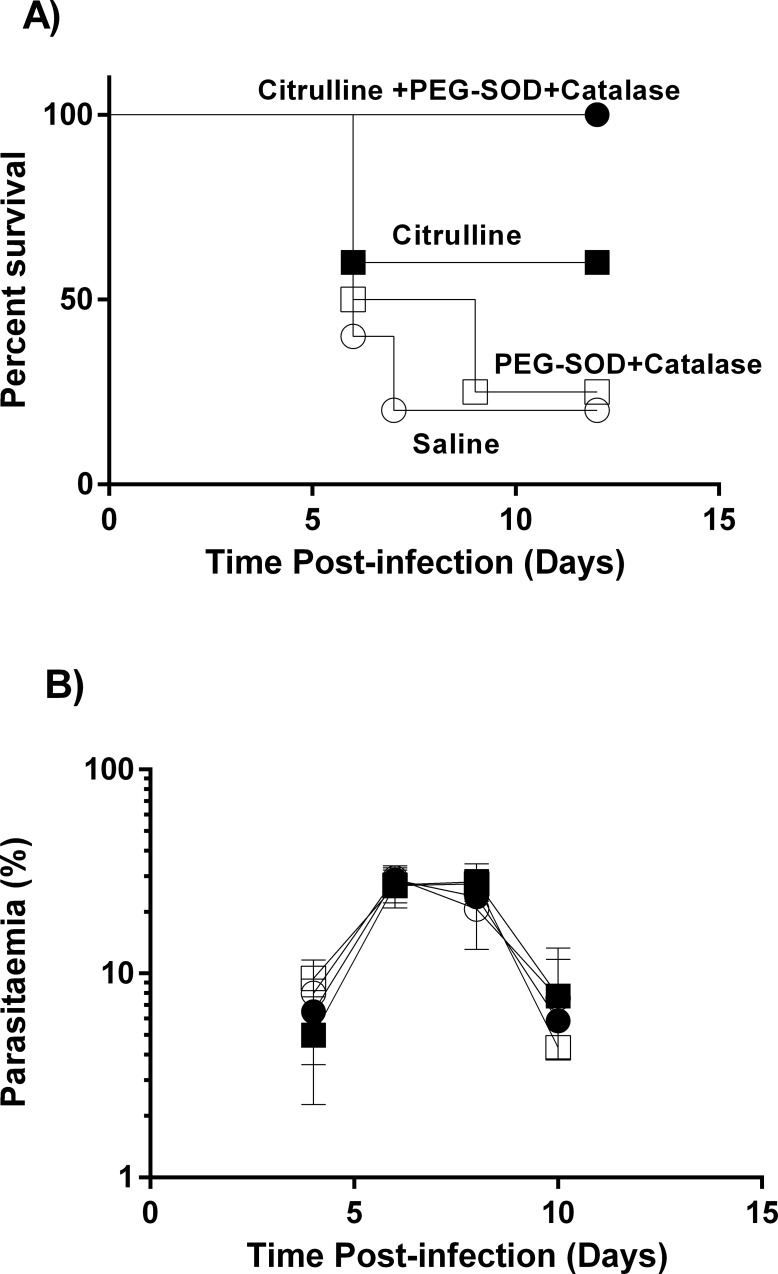
Citrulline plus SO scavenging by PEG-SOD+PEG-CAT provides complete protection. Four groups (n = 5) of mice were injected i.v. with PbA. (**A):** survival, (**B):** parasitemia (mean % ± stderr) timecourse of the groups of mice injected with (i) citrulline, (ii) PEG-SOD+PEG-CAT, (iii) citrulline plus PEG-SOD+PEG-CAT, or (iv) saline from day 4 PI with quinine drug rescue on day 6PI. This experiment was repeated with similar results.

To determine whether citrulline injection, SO scavenging, or both function as an adjunctive therapy to rescue from eCM, we injected ip from day 4PI (i) citrulline or (ii) vehicle control twice daily, (iii) PEG-SOD+PEG-CAT (SO scavenger) or (iv) Vehicle control once daily, or (v) citrulline plus SO scavenger. The mice were injected with quinine once daily for 5 consecutive days beginning on day 6PI to kill parasites. The citrulline and SO scavenger treated mice exhibited some (~10 and ~40% respectively) but not significant protection from eCM mortality, but the combination of citrulline with SO scavenger significantly (p<0.05) protected all recipients (Fig 4A). The parasitemia was similar in all groups of mice (Fig 4B). The partial protection in S3 Fig was obtained with SO scavenger prophylaxis (daily injection from day 1PI) whereas the result in Fig 4 was obtained with SO scavenger adjunctive therapy. This result indicates that co-adjunctive treatment of citrulline plus SO-scavenger elicits significant (p<0.05) protection from eCM and that the effect is additive compared with individual treatments.

## Discussion

Profound hypoargininema leads to impaired nitric oxide bioavailability during SM pathogenesis[1–4]. Low dose (12g) arginine injections into UM and moderately ill patients are well tolerated and restore NO bioavailability as measured by reactive hyperemia[1–4]. The inability of infused low dose arginine to restore NO bioavailability in severely ill malaria patients indicates that additional factors are preventing the restoration of plasma arginine levels and consequently the coupling of NOS enzyme with cationic amino acid transporter (CAT) [34]. The arginine paradox states that the concentration of plasma (extra-EC) arginine determines NO production by eNOS even though intra-EC arginine levels are normal [35]; this paradox is due to the coupling between CAT and eNOS, providing the arginine substrate for eNOS rather than intra-EC arginine. Because hypoargininemia is significantly increased in SM compared with UM patients and arginase activity is increased in SM, it is likely that infused arginine is converted by plasma arginase into ornithine and so is not available to be transported into EC via CAT and converted into NO. Infused citrulline is rapidly converted to arginine, so it similarly restores plasma arginine levels. The advantage of infused citrulline over arginine is that citrulline inhibits arginase, and exhibits decreased toxicity in cardiovascular disease and maintains plasma arginine levels for longer than infused arginine [18], suggesting that citrulline may be an improved nutraceutical or adjunctive therapy for CM over arginine.

In eCM, hypoargininemia is an early (day 4PI) event prior to the onset of eCM and coincided with elevated plasma ornithine, which suggests that arginine may be consumed by arginase. Indeed, arginase activity is elevated in eCM plasma. The levels of plasma ornithine decline from day 4 PI to day 6 PI (eCM), suggesting that prolonged and profound hypoargininemia may contribute to the decline in ornithine levels. That is, a lack of substrate for arginase may prevent the build up of product (ornithine) at eCM. Similar time course studies cannot be undertaken ethically in humans, but both CM patients and eCM mice exhibit decreased levels of plasma ornithine [36].

Our observation that ip injection of both citrulline and arginine rapidly (within 15min) restores plasma arginine levels to normal in mice on day 5 PI with *P*. *berghei* and maintains the levels for >4h indicates that injection of either amino acid ameliorates malarial hypoargininemia. As reported by Cobbold et al. [36], D2-labeled citrulline infusion confirms that this amino acid is converted to arginine and restores arginine levels to normal when injected on day 5PI. The levels of ornithine, the end-product of arginase, increases rapidly in the arginine-injected animals compared with citrulline-injected animals, suggesting that plasma arginase is converting infused arginine into ornithine. Citrulline injection ip significantly inhibits arginase activity compared with vehicle controls, suggesting that the lower slope of increase in ornithine may be due to citrulline inhibition of arginase. These observations suggest that citrulline infusion may be better than arginine for eCM because citrulline restores also plasma arginine levels and by inhibiting plasma arginase may allow increased plasma arginine uptake by eNOS coupled cationic amino acid (arginine) transporter for subsequent conversion to NO. Ornithine inhibits the arginine transporter [37, 38]; thus elevated ornithine induced by arginine infusion into infected animals likely inhibits the arginine transporter coupled to eNOS, thereby exacerbating eNOS uncoupling. Uncoupled eNOS produces SO rather than NO [35]. Arginine restoration also improves RBC deformability [39], which may be a key factor in vascular plugging during human CM [40] and eCM [41].

Our results indicate that citrulline injected prophylactically as a nutraceutical provides complete protection from eCM mortality. Because parasitemia is similar in citrulline-injected and vehicle control groups, the protection is not due to an effect on parasite replication, such as the restoration of NO production that is parasiticidal. This conclusion agrees with our previous reports that NO has no detectable effect on parasite viability in part due to the RBC hemoglobin that surrounds the developing parasite and scavenges any free NO [12]. The citrulline nutraceutical abrogated the breakdown of the BBB during eCM, and maintained brain metabolic activity as assessed by PET. The breakdown of the BBB and brain swelling are important components of CM pathogenesis in humans [42, 43].

The ip injection of arginine into the peritoneal cavity resulted in marked lesions at the site of injection and flaking of the skin. The reaction is elicited about day 3 PI, resulting in the discontinuation of injections and euthanization of the affected animals. We were therefore unable to test arginine nutraceutical administered in defined doses to the peritoneal cavity. We therefore added both arginine and citrulline to the drinking water. Both amino acids significantly (p<0.05) protected animals from eCM mortality, however, the arginine treated group had significantly lower parasitaemia when compared to citrulline treated or vehicle control groups.

This nutraceutical study suggests that differences in diet may affect whether an individual develops CM. Additional studies are required to determine whether citrulline or arginine nutraceutical will translate into prophylaxis for.Diet may therefore be an additional factor explaining why limited numbers of people infected with *P*. *falciparum* develop CM. Awasthi et al. [44] reported that citrulline inhibits *in vitro* replication of *P*. *falciparum*, suggesting an additional beneficial effect of a citrulline nutraceutical. We, however, did not observe any effect of citrulline parasitemia and hence parasite replication *in vivo* despite hypercitrullinemia. In almost half the experiments, we observed significantly (p<0.05) decreased parasitemia in eCM mice with oral arginine compared with water controls.

Most evidence currently suggests that SO role in experimental malaria may be limited. Mice deficient in NOX subunits (pHox47 and pHox67) exhibit similar *P*. *chabaudi* parasitemia as WT controls [45], indicating that SO is not limiting parasite replication *in vivo* in mice. Dr. Hunt’s group reported that NADPH-oxidase- (gp91) and glutathione peroxidase-1 knockout mice are not protected from eCM, exhibit similar parasitemia as WT controls, and that brain markers of oxidative tissue damage (protein carbonyls, 3,4-dihydrophenylalanine, o-tyrosine, and dityrosine) are similar in eCM (PbA) mice, non-eCM mice (Pb K173; PbK), and uninfected controls [31–33]. Brain urate levels are similar in eCM and non-eCM mice, indicating xanthine oxidase (XO) dependent purine breakdown is not specific to eCM [33]. These data indicate that NOX- and XO-derived SO does not affect parasitemia or the development of eCM. Moreover, a small scale trial of N-acetyl cysteine to scavenge SO failed to show any benefit. However, malaria patients in East Asia exhibit increased urinary F2-isoprostane metabolites, a marker for oxidative stress [46], suggesting SO may function in SM pathogenesis.

Our studies show that PEG-SOD+PEG-CAT significantly (p<0.05) protects against eCM mortality when injected prophylactically from day 1 PI (S3 Fig) and exhibited decreased (not significantly) mortality when injected as adjunctive therapy (Fig 4). Because the injected SO scavenger does not affect parasitemia, we conclude that SO is a key component of the pathogenic process rather than parasiticidal. As described above, the pathogenic SO is unlikely derived from NOX or XO and so may be derived from uncoupled eNOS or free hemoglobin during eCM [11, 12]. Indeed, eNOS [14] is uncoupled during eCM and tetrahydrobiopterin, a cofactor for NOS, levels are low in the urine of SM and CM patients, suggesting that eNOS is a source for SO. Free hemoglobin is elevated during eCM and in patients with SM, suggesting that it may be an additional source of SO. Whichever is the SO source, scavenging of SO and restoration of NO bioavailability are important to ameliorate eCM pathogenesis and mortality in mice. Our observation that either SO scavenging or citrulline as an adjunctive therapy provides partial protection from eCM mortality supports this contention. The complete protection from eCM mortality elicited by both SO scavenging and citrulline as adjunctive therapy indicates synergy of SO scavenging and amelioration of hypoargininemia.

## Supporting information

S1 Fig Disruption of levels of arginine, citrulline, and ornithine (urea cycle amino acids) during the course of eCM. (**A):** Summary of the urea cycle showing primary organ-specific location of the cycle. (**B):** Arginine, citrulline, and ornithine amino acid levels during the course of eCM. Groups (n = 5) of mice were injected i.p. with PbA and plasma obtained on day 0 (uninfected), 4 (patent), and 6 (eCM) PI. The amino acids measured by HPLC are reported as mean ± stderr. : p<0.05. This experiment was repeated with similar results.
(TIF)

S2 Fig Partial restoration of plasma arginine but elevated plasma citrulline and ornithine in citrulline nutraceutical mice on day 6 PI compared with saline controls with eCM. Groups (n = 5) of mice were injected i.p. with PbA and plasma obtained on day 0 (uninfected), 4 (patent), and 6 (eCM) PI. Mice are injected ip twice daily with citrulline nutraceutical or saline control from day 1 PI, and plasma isolated at selected timepoints. The amino acids (A) arginine, (B) citrulline, (C) ornithine, and (D) glutamine measured by HPLC are reported as mean ± stderr. : p<0.05. This experiment was repeated with similar results. **(E)**: Average pixel intensity PET brain images in groups (n = 5) mice infected with PbA and treated with citrulline nutraceutical or saline and compared with uninfected controls. This experiment was performed once. Arginine injection ip resulted in toxicity in the first experiment that was then terminated and not repeated. : p<0.05.
(TIF)

S3 Fig SO scavenging by PEG-SOD+PEG-CAT as prophylaxis from day 1PI partially protects against eCM compared with PEG-CAT. Groups (n = 5) of mice of mice were injected i.p. with PbA on day 0PI. (A) Survival, and (B) parasitemia of mice were injected with either PEG-SOD+PEG-CAT or vehicle control daily from day 1 PI. *: p<0.05. This experiment was repeated with similar results.
(TIF)

S4 Fig No improvement in thrombocytopenia in eCM mice with citrulline administered as a nutraceutical. Groups (n = 5) of mice of mice were injected i.p. with PbA on day 0PI. (A) Thrombocytopenia, and (B) parasitemia in mice injected with either citrulline or vehicle control daily from day 1 PI. No significant difference (p>0.05) between the groups. This experiment was repeated with similar results.
(TIF)

S1 Table Partial restoration of chemokine levels in eCM mice with citrulline administered as a nutraceutical. Groups (n = 5) of mice of mice were injected i.v. with PbA on day 0PI. Mice were treated twice daily with Citrulline or vehicle control (i.p) beginning on day 1PI; uninfected, untreated mice were used as controls. On day 6PI, plasma was obtained and analyzed for chemokines by MyriadRBM (Austin,TX). p<0.05 Citrulline vs Saline; p<0.05 Citrulline vs Uninfected; p<0.05 Saline vs Uninfected.
(DOCX)

S2 Table No improvement in coagulation markers in eCM mice with citrulline administered as a nutraceutical. Groups (n = 5) of mice of mice were injected i.v. with PbA on day 0PI. Mice were treated twice daily with Citrulline or vehicle control (i.p) beginning on day 1PI; uninfected, untreated mice were used as controls. On day 6PI, plasma was obtained and analyzed for chemokines by MyriadRBM (Austin,TX). No significant difference was observed between any of the groups.
(DOCX)
