# Supplementary material for: Citrulline protects mice from experimental cerebral malaria by ameliorating hypoargininemia, urea cycle changes and vascular leak
Source: PLoS One. 2019 Mar 8;14(3):e0213428. doi: 10.1371/journal.pone.0213428 (PMC6407779; doi:10.1371/journal.pone.0213428)
Supplement: S1 Table — Groups (n = 5) of mice of mice were injected i.v. with PbA on day 0PI. Mice were treated twice daily with Citrulline or vehicle control (i.p) beginning on day 1PI; uninfected, untreated mice were used as controls. On day 6PI, plasma was obtained and analyzed for chemokines by MyriadRBM (Austin,TX). p<0.05 Citrulline vs Saline; p<0.05 Citrulline vs Uninfected; p<0.05 Saline vs Uninfected. (DOCX) [file pone.0213428.s005.docx]

|  | **Uninfected (n=3)** | **Citrulline (n=5)** | **Saline (n=5)** |
| --- | --- | --- | --- |
| **VCAM-1 ⚫◆◼** | 833.67 ± 14.57 | 1684 ± 216.75 | 2796 ± 171.26 |
| **TNF-α ⚫◼** | 0.03 ± 0.01 | 0.08 ± 0.01 | 0.13 ± 0.03 |
| **TMP1 ◼** | 0.83 ± 0.29 | 2.60 ± 0.79 | 4.38 ± 2.37 |
| **MIP1α ◼** | 3.43 ± 1.25 | 4.50 ± 0.67 | 5.46 ± 0.70 |
| **MIP3β ⚫◼** | 1.05 ± 0.33 | 1.36 ± 0.49 | 2.56 ± 0.93 |
| **MMP9 ⚫◼** | 32 ± 5.29 | 58.6 ± 19.03 | 97.4 ± 19.58 |
| **Lymphotactin ⚫◆◼** | 87.33 ± 14.57 | 255.8 ± 69.52 | 392.8 ± 62.22 |
| **IL-18 ⚫◼** | 7.43 ± 1.33 | 76.4 ± 34.86 | 188.8 ± 54.75 |
| **MCSF1 ⚫◼** | 58 ± 0.96 | 6.38 ± 0.63 | 8.72 ± 0.53 |

# Table S1:Partial restoration of chemokine levels in eCM mice treated with Citrulline.
