# Supplementary material for: Citrulline protects mice from experimental cerebral malaria by ameliorating hypoargininemia, urea cycle changes and vascular leak
Source: PLoS One. 2019 Mar 8;14(3):e0213428. doi: 10.1371/journal.pone.0213428 (PMC6407779; doi:10.1371/journal.pone.0213428)
Supplement: S2 Table — Groups (n = 5) of mice of mice were injected i.v. with PbA on day 0PI. Mice were treated twice daily with Citrulline or vehicle control (i.p) beginning on day 1PI; uninfected, untreated mice were used as controls. On day 6PI, plasma was obtained and analyzed for chemokines by MyriadRBM (Austin,TX). No significant difference was observed between any of the groups. (DOCX) [file pone.0213428.s006.docx]

|  | **Uninfected (n=3)** | **Citrulline (n=5)** | **Saline (n=5)** |
| --- | --- | --- | --- |
| **Factor VII (ng/ml)** | 24 ± 0 | 21.2±4.15 | 26±4.47 |
| **Fibrinogen (mg/ml)** | 81700 ± 56623 | 68220±8005 | 86760±9735 |
| **Tissue Factor (ng/ml)** | 5.20 ± 0.52 | 5.28±0.85 | 6.04±1.04 |
| **vWF (ng/ml)** | 310±48 | 341.2±71 | 426±48.7 |

# Table S2: No improvement in coagulation markers in eCM mice with citrulline administered as a nutraceutical.
